# Effects of Asian dust-derived particulate matter on ST-elevation myocardial infarction: retrospective, time series study

**DOI:** 10.1186/s12889-020-10067-y

**Published:** 2021-01-07

**Authors:** Suji Lee, Whanhee Lee, Eunil Lee, Myung Ho Jeong, Seung-Woon Rha, Chong-Jin Kim, Shung Chull Chae, Hyo-Soo Kim, Hyeon-Cheol Gwon, Ho Kim

**Affiliations:** 1 Institute of Health and Environment, Seoul National University, Gwanak-ro, Seoul, 08826 Republic of Korea; 2 Department of Biostatistics and Epidemiology, Graduate School of Public Health, Seoul National University, Gwanak-gu, Seoul, 00826 Republic of Korea; 3 Department of Preventive Medicine, College of Medicine, South Korea University, Anam-ro, Seoul, 03080 Republic of Korea; 4 Chonnam National University Hospital, Jebong-ro, Gwangju, 61469 South Korea; 5 Korea University Guro Hospital, Gurodong-ro, Seoul, 08308 South Korea; 6 Kyunghee University Hospital at Gangdong, Gangdong-gu, 05278 Seoul, Republic of Korea; 7 Department of Internal Medicine, Kyungpook National University Hospital, Jung-gu, 41940 Daegu, Republic of Korea; 8 School of Medicine, Kyungpook National University, Jung-gu, 41940 Daegu, Republic of Korea; 9 Department of Internal Medicine, Seoul National University Hospital, Jongno-gu, 03080 Seoul, Republic of Korea; 10 Samsung Medical Center, Sungkyunkwan University School of Medicine, Gangnam-gu, 06351 Seoul, Republic of Korea

**Keywords:** Particulate matter, Myocardial infarction, Asian dust, Air pollution, Health effect

## Abstract

**Background:**

Dust storms affect human health by impairing visibility and promoting interactions with microscopic organisms, such as bacteria and fungi. Although ST-elevation MI (STEMI) and non-ST-elevation MI (NSTEMI) differ mechanistically, few studies have investigated the incidence of cardiovascular diseases according to infarction type; these studies have yielded inconsistent findings. This study aimed to examine whether PM size (< 2.5 μm (PM_2.5_) and < 10 μm (PM_10_)) modifies the effect of Asian dust on acute myocardial infarction (AMI), with separate analyses for STEMI and NSTEMI.

**Methods:**

MI-related data from 9934 emergency visits were collected from the Korea AMI Registry from 2005 to 2017. Asian dust events were defined as days with visibility of ≤10 km. Generalized linear models were used to analyze data with natural cubic splines. To examine potential modifiers, analyses were stratified by age, smoking status, and body mass index (BMI).

**Results:**

No significant associations were observed between Asian dust and AMI. By adjusting for different lag structures, a significant effect was exclusively observed in STEMI. For moving average lags, the largest value at lag 5 (relative risk [RR] 1.083; 95% confidence interval [CI], 1.007–1.166) for single and lags 0–7 (RR 1.067; 95% CI: 1.002–1.136) was observed for PM_2.5_; for PM_10_, the largest significant effect was observed at lag 4 (RR 1.075; 95% CI: 1.010–1.144) for single and lags 0–7 (RR 1.067; 95% CI: 1.002–1.136). RRs were significantly higher in < 65-year-olds than in ≥65-year-olds. Additionally, RRs between the BMI < 25 and BMI ≥ 25 groups were not different; statistically significant effects were observed for concentration at lags 0–5 (RR: 1.073; 95% CI: 1.002–1.150) and lags 0–6 (RR: 1.071; 95% CI: 1.001–1.146) in the BMI < 25 group. A negative exposure-response association was observed between daily average visibility-adjusted PM and STEMI and daily average visibility-adjusted PM in < 65-year-olds.

**Conclusions:**

Reducing PM_2.5_ and PM_10_ emissions, particularly during the days of Asian dust, may be crucial and reduce STEMI and AMI incidence among < 65-year-olds. These results indicate that the Asian dust alarm system needs revision to protect vulnerable populations.

## Background

Dust storms affect human health by impairing visibility and promoting interactions with microscopic organisms, such as bacteria and fungi [1]. They have recently been associated with diseases and adverse health effects and are being recognized as a health risk. Considerable epidemiological research has been published on cardiovascular impacts of dust storms in East Asian countries, such as China [2] and Japan [3, 4], as well as southern Europe [5] and North America [6]. These adverse health effects include asthma, respiratory diseases, cardiovascular diseases, and mortality [2, 4–6]. By contrast, some studies have observed no association between dust storms and cardiovascular disease in either emergency department or hospital admissions [7].

There is an increasing evidence that increased exposure to particulate matter (PM) leads to cardiovascular disease, particularly among patients with a history of myocardial infarction (MI) [8–11]. Although ST-elevation MI (STEMI) and non-ST-elevation MI (NSTEMI) differ mechanistically [12], few studies have investigated the incidence of cardiovascular diseases according to infarction type; these studies have yielded inconsistent findings. A New York-based study initially suggested that exposure to PM size < 2.5 μm (PM_2.5_) increased the risk of STEMI, unlike NSTEMI [9]. Moreover, studies from Belgium and Utah (US) reported consistent results [8, 11]. Conversely, other studies failed to demonstrate the effect of PM_2.5_ on STEMI risk [13, 14].

The European project “MED-PARTICLES” underscored that the adverse health effect of the desert component of PM can be affected by concentrations of anthropogenic-derived PM size **<** 10 μm (PM_10_) [15]. However, past approaches that investigated the health effects of PM and Asian dust separately analyzed each component, whereas few researches simultaneously studied the two effects. Additionally, various natural crustal elements (Ca^2+^, Mg^2+^, Na^+^, K^+^), anthropogenic pollutants (Pb, As, NO_x_), microorganisms, fungal spores, chemical components, and gaseous pollutants can also be mixed and carried in the dust [16]. It is possible that dust event-associated health effects involving PM are different from those associated with dust events alone [17]; therefore, it is necessary to research the health effects of dust storms with PM.

Therefore, this study aimed to evaluate the association between AMI and Asian dust with PM, and assess the variation of the association according to infarction type (STEMI and NSTEMI), age group, body mass index (BMI), and smoking status.

## Methods

### Data collection

The KAMIR is a prospective study of approximately 37,880 patients in 57 general emergency rooms of Korean hospitals that collects data using a standardized case report form. The rationale and design of this nationwide mandatory registration have been previously explained [18]. This study included patients from the KAMIR who presented with AMI to the Seoul hospital emergency rooms between 2005 and 2017 and for whom PM_2.5_ data were available. Of these, patients with inadequate information were excluded. The first medical contact time was defined as the time of the primary manifestation of AMI symptoms. In addition, stratified analyses were performed by age, smoking status, and MI status (STEMI and NSTEMI).

Climate data were obtained from the Korean Meteorological Administration, including those on the daily mean temperature, humidity, sea level pressure, and visibility from 2005 to 2017. Based on previous studies, we defined Asian dust events as days when visibility was < 10 km [3, 4]. PM_10_ data were obtained from the National Institute of Environmental Research in Korea, whereas PM_2.5_ data were collected from 27 monitoring sites managed by the Seoul Research Institute of Public Health and Environment.

### Statistical analysis

The time-series data for this ecological study design were analyzed using generalized linear models to accommodate the nonlinear relationships of emergency visits for AMI. Natural cubic spline functions were used to consider non-linearity for time and weather variables. In the basic model, single lag day effects of Asian dust were examined from the current day (lag 0) to the sixth day, adjusting for meteorological effects that could affect AMI, including two-day moving average of temperature, current day relative humidity, and sea level pressure (lag 0), as covariates. In the evaluation of the hypothesis that PM_2.5_ and PM_10_ would enhance MI risk on Asian dust days, single- and moving average lags of PM_2.5_ and PM_10_ were included in the basic model. The largest lag days of Asian dust in the basic model were used in the PM-adjusted model. Similarly, stratified analyses were conducted by age, smoking status (never smoking/past smoking/current smoking), and obesity to evaluate the potential effect of the association between Asian dust and AMI. BMI was categorized into two categories based on the standard definition as follows [19]: normal/underweight (BMI < 25 kg/m^2^) and overweight/obese (BMI ≥25 kg/m^2^).

Finally, sensitivity analyses were performed to assess the validity of the findings by modified different degrees of freedom for time trends and various temperature lag structures. Additionally, because higher relative humidity could plausibly affect visibility, models were repeated to include interaction terms between daily mean visibility and an indicator variable of high level of humidity days, wherein relative humidity was higher than 70% or 80%, respectively. All statistical analyses were performed using R software 3.4.0 (Vienna, Austria).

## Results

Table 1 presents baseline characteristics of study participants. STEMI and NSTEMI occurred in 5366 and 4497 patients, respectively. Regarding STEMI, there were 63.8% patients aged ≥65 years, and 78.8% were male. Regarding NSTEMI, there were 70.78% patients aged ≥65 years, and 68.4% were male. STEMI patients were more likely to be current smokers (44.3%) than NSTEMI patients (33.5%).

**Table 1:** Characteristics of patients with myocardial infarction in Korea during 2005–2017 (*N* = 9934)

Characteristic	STEMI		NSTEMI	
	***N*** = 5366	54.0%	***N*** = 4497	45.2%
**Age**				
< 65 years (*n* = 3277)	1893	35.3	1288	28.7
≥ 65 years (*n* = 6657)	3424	63.8	3183	70.8
Mean ± SD	70.0 ± 14.2		72.4 ± 14.1	
**Male** (*n* = 7326)	4194	78.3	18,204	68.4
**Smoking**				
Never	2111	39.1	2165	49.3
Ex	811	14.9	7,57	17.2
Current	2323	44.3	1474	33.5
Unknown history	121		101	
**Medical history**				
Hypertension	2577	49.4	2583	58.5
Diabetes mellitus	1391	26.7	1486	33.8
Dyslipidemia	653	12.5	708	16.1
**BMI**				
Normal weight (BMI < 25 kg/m^2^)	2852	53.2	2468	54.9
Overweight (BMI ≥25 kg/m^2^)	1814	33.9	1559	34.7

(Missing = 71). *STEMI* ST-elevation myocardial infarction, *NSTEMI* non-ST-elevation myocardial infarction, *STD* standard deviation, *BMI* body mass index

Table 2 shows the daily mean concentrations of air pollutants and weather conditions in Seoul with and without Asian dust events. Concentrations of pollutants, excluding O_3_, were higher during Asian dust days than during days without Asian dust events. We found that Pearson’s correlation coefficients of PM concentrations was more strongly related with visibility than were other air pollutants such as NO_2_, SO_2_, O_3_, and CO (Additional file 1). In addition, after excluding relative humidity higher than 80%, visibility retained a high negative correlation with PM.

**Table 2:** Mean level of air pollution on Asian dust- (visibility ≤10 km) and non-Asian dust days

	Mean level on Asian dust days (*n* = 1301)	Mean level on non-Asian dust days (*n* = 2875)	*P* value
*Air pollutant concentrations*			
CO (ppm)	0.8 ± 0.4	0.6 ± 0.3	< 0.0001
O_3_ (ppb)	31.6 ± 19.5	32.6 ± 16.9	0.102
NO_2_ (ppb)	38.5 ± 12.9	31.3 ± 11.7	< 0.0001
SO_2_ (ppb)	6.1 ± 2.7	5.1 ± 1.6	< 0.0001
PM_10_ (μg/m^3^)	70.6 ± 41.8	42.3 ± 20.0	< 0.0001
PM_2.5_ (μg/m^3^)	39.1 ± 17.1	22.1 ± 8.9	< 0.0001
*Weather conditions*			
Mean temperature (°C)	13.76 ± 9.22	11.95 ± 11.15	< 0.0001
Relative humility (%)	70.9 ± 12.08	55.26 ± 13.36	< 0.0001

*CO* carbon monoxide, *O*_*3*_ ozone, *NO*_*2*_ nitrogen dioxide, *SO*_*2*_ sulfur dioxide, *PM*_*10*_ particulate matter with a median aerodynamic diameter ≤ 10 μm; ppb, PM_2.5_, fine particulate air matter with an aerodynamic diameter < 2.5 μm; ppb, parts per billion; ppm, parts per million

**Fig. 1:**
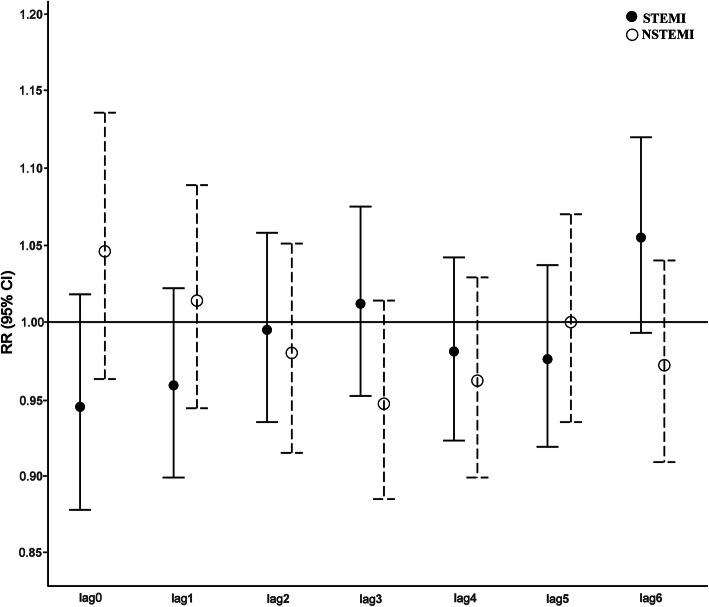
RRs^1^of ST elevation myocardial infarction (STEMI) and non-STEMI (NSTEMI) associated with dust days. Single lags are shown for the current day (lag 0) to 6 days (lag 6). Comparison with non-dust days according to lag days in 2005–2017 (Relative risks with 95% confidence interval)

Figure 1 presents the relative risks (RRs) of STEMI and NSTEMI associated with dust days, according to lag days. The largest effect was observed in STEMI patients at the lag 6 concentration (RR, 1.055; 95% confidence interval [CI], 0.993–1.120), whereas that in NSTEMI patients was observed on the current day (RR, 1.046; 95% CI, 0.963–1.136). However, the values were not significant. While evaluating the effect of PM, significant associations were observed between the RRs of NSTEMI and PM_10_ and between the subgroup of NSTEMI patients aged ≥65 years and PM_2.5_ (Additional file 2).

**Fig. 2:**
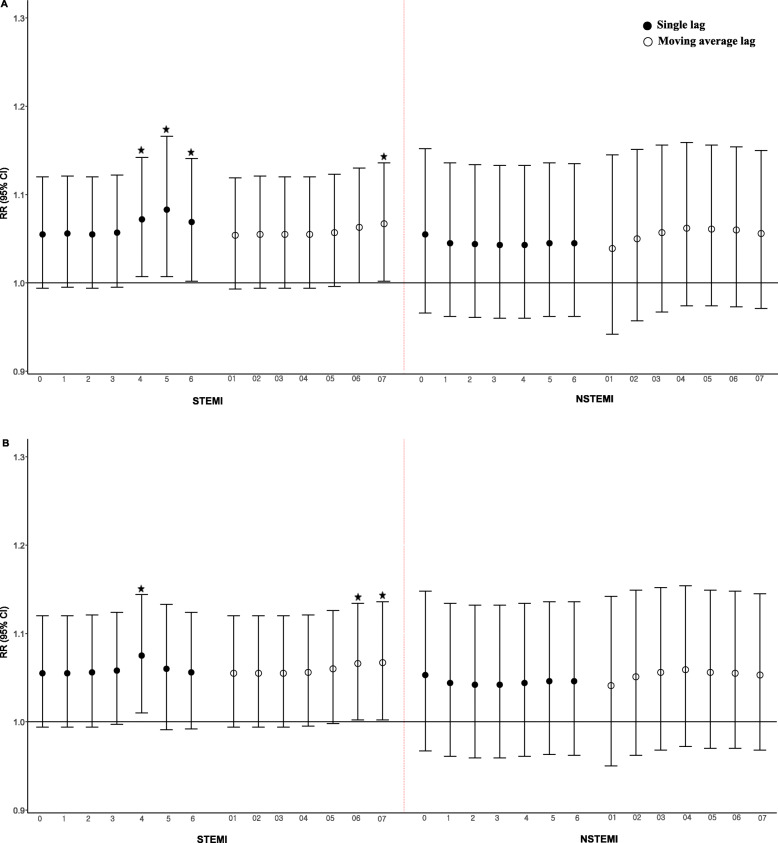
RRs^1^ of Asian dust and acute myocardial infarction stratified by STEMI and NSTEMI Adjustment for (**a**) PM_2.5_ and (**b**) PM_10_. The current day (lag 0) to six days (lag 6) were for single lags, and for the current day and one day before day (lag 01) to seven days (lag 07) were for moving average lags. Relative risks were adjusted for the two-day moving averages of temperature, sea level pressure, current day relative humidity long-term trends, and seasonality. Six-day (lag 6) lags of Asian dust were used for STEMI and current day lag (lag 0) of Asian dust was used for NSTEMI. * Significance at *p* < 0.05

Figure 2 illustrates the association between Asian dust exposure and onset of STEMI and NSTEMI, after adjustment for PM_2.5_ and PM_10_, by different lag structures. For STEMI, RRs increased after adjustment for PM_2.5_ and PM_10_ (for both single- and moving average lags). On considering PM_2.5_ in the model, the most significant effect was observed at lag 5 (RR 1.083; 95% CI, 1.007–1.166) for single lags and lags 0–7 (RR 1.067; 95% CI: 1.002–1.136) for moving average lags. In the course of controlling for PM_10_ in the model, the largest significant effect was observed at lag 4 (RR 1.075; 95% CI: 1.010–1.144) for single lags and lags 0–7 (RR 1.067; 95% CI: 1.002–1.136) for moving average lags. On the contrary, Asian dust exposure had no significant risk for the onset of NSTEMI for both single- and moving average lags.

**Fig. 3:**
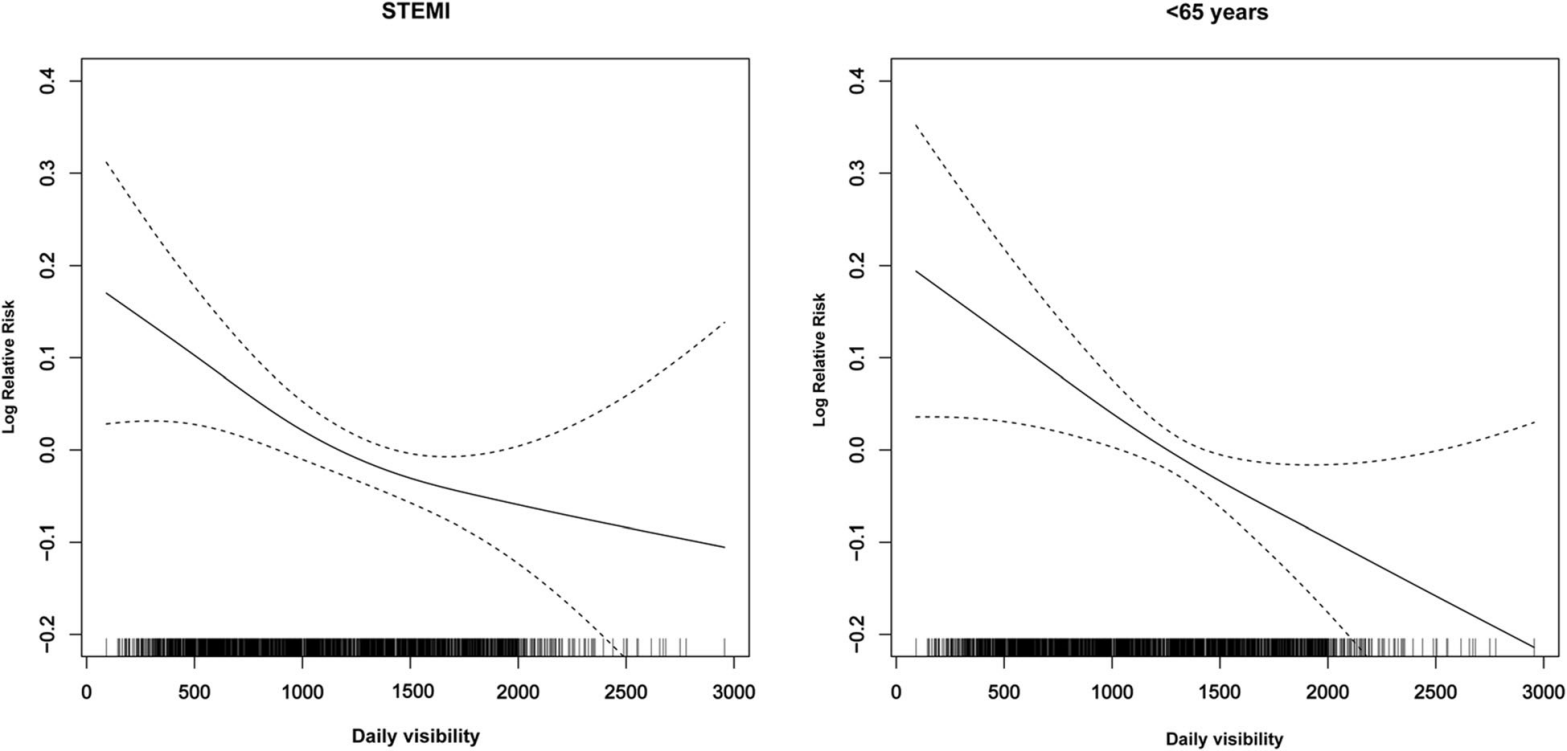
Dose-response relationship between daily visibility and STEMI (left) in those aged < 65 years (right) after adjustment for PM_2.5._ The x-axis represents the daily visibility in the six previous days. The y-axis represents the relative risk, after controlling for PM_2.5_ concentration on lag 5, long-term trends, seasonality, two-day moving average of temperature, sea level pressure, and current day relative humidity

**Fig. 4:**
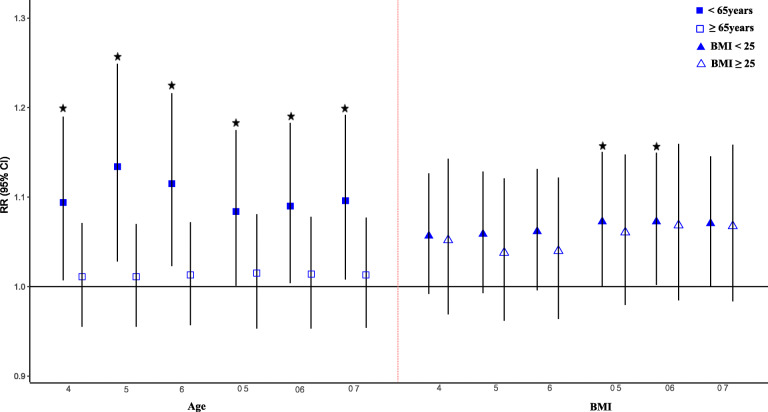
RRs^1^ of acute myocardial infarction associated with Asian dust, stratified by age and body mass index (BMI) after adjusting for PM_2.5._ RRs were adjusted for long-term trends, seasonality, two-day moving average of temperature, sea level pressure, and current day relative humidity. Six-day (lag 6) lags and two-day (lag 2) lags of Asian dust were used for age < 65 years and ≥ 65 years, respectively. Two-day (lag 2) lags and five-day (lag 5) lags of Asian dust were used for BMI < 25 and BMI ≥25 kg/m2, respectively. * Significance at p < 0.05

Figure 3 illustrates clearly negative and linear exposure-response relationships between daily visibility at lag 5 and STEMI in those aged < 65 years. As PM_2.5_ demonstrated a larger impact of Asian dust on the incidence of AMI, using an age group- and BMI-based stratification analysis, the effect of PM_2.5_ was further examined (Fig. 4). A significantly greater risk was observed in the < 65-year-old age group for single lags at lag 5 (RR 1.134; 95% CI: 1.028–1.249) and moving average lags at lags 0–5 (RR, 1.083; 95% CI, 1.007–1.166). RRs between the BMI < 25 kg/m^2^ and BMI ≥25 kg/m^2^ groups were not different; however, significant effects were observed for the PM_2.5_ concentrations at lags 0–5 (RR, 1.073; 95% CI, 1.002–1.150) in the BMI < 25 kg/m^2^ group. When PM_10_ was considered in the model, a similar pattern was observed with PM_2.5_ for both age group and BMI; however, a significant effect was exclusively observed in < 65-year-old age group (Additional file 3).

Although our results did not achieve statistical significance owing to the small sample size, RRs were higher in the past smoking group than in the never smoking and current smoking groups in the PM_2.5_ models (Additional file 4). Data in Additional file 5 suggest robust associations between Asian dust and STEMI after adjustment for PM_2.5_ (Additional file 5).

## Discussion

This study clarifies how PM impacts the effects of Asian dust on AMI incidence. The data showed no significant association between Asian dust and AMI at any lag; however, the association became significant after adjustment for PM_2.5_ and PM_10_, and thus, PM might have increased the effect of Asian dust on AMI risk. More importantly, both PM_2.5_ and PM_10_ enhanced the impact of Asian dust on increased risk for STEMI, but not for NSTEMI; this is consistent with findings of prior studies [8, 10]. In addition, RRs for AMI were significantly higher in the < 65-year-old and BMI < 25 groups than in the ≥65-year-old and BMI ≥25 kg/m^2^ groups. In particular, this is the first study to show the relationship between daily average visibility-adjusted PM concentrations and STEMI. The results suggest the presence of an approximately negative linear exposure-response curve between visibility and STEMI; moreover, these results suggest that the visibility degradation’s effects on the incidence of STEMI varied according to PM_2.5_.

Evidence of the risk of MI with short-term exposure to PM has been widely reported. A meta-analysis by Mustafic et al. that combined estimates of 34 eligible research suggested that short-term exposure to PM increased the risk of MI [20]. Most studies have focused on AMI as the health outcome; however, interest has shifted to specific subsets of AMI more recently. For instance, Gardner et al. provided information on the relationship between PM_2.5_ exposure STEMI and, rather than NSTEMI [9]. Consistent with this, a recent study in China similarly showed that PM_2.5_ may increase the risk of STEMI but not NSTEMI [10]; these are consistent with the present findings.

Numerous studies have reported the health effects of Asian dust exposure; however, relatively few studies have focused on AMI onset, possibly due to insufficient sample size regarding AMI incidence within the relevant geographic area. Matsukawa et al. suggested that exposure to Asian dust a few days before admission is associated with the incidence of AMI [4]; thus, Asian dust may be a trigger of AMI. Nevertheless, there is still inadequate understanding on how PM affects the association of AMI with Asian dust that is further complicated by inconsistent reports. For example, Crooks et al. observed associations of dust storms and PM_10_ with AMI in the US [6]; but not of dust storms with PM_2.5_. Furthermore, Neophytou et al. reported a study in Cyprus wherein a positive effect of dust day PM_10_ on cardiovascular mortality was observed; however, the association steadily decreased on non-dust days [21]. For the effects of PM_2.5_, Kim et al. reported that cardiovascular mortality was significantly associated with PM_2.5_ during Asian dust days in Korea [22]. Similarly, a recently conducted Japanese study by Kojima et al. observed that Asian dust increased the risk of AMI after controlling for PM_2.5_ [3]. However, contrary to this study’s finding, the association with Asian dust was reported to be more pronounced for NSTEMI than for STEMI. The effects of PM_2.5_ on different subgroups of AMI may explain the inconsistent results observed in earlier studies.

Another important finding of the present analysis was that the effects of Asian dust exposure, alongside short-term PM exposure, were substantially dependent on age and BMI. Contrary to comparable epidemiological studies wherein larger effects were observed in older individuals [3, 23], a stronger impact on the AMI onset was observed in the younger age group. The reason could be the participation of young people in many outdoor activities. Consistently, Chen et al. reported that the risk of emergency hospital visits for PM_10_ and PM_2.5_ was slightly higher in the 46–64-year-old age group than in the ≥65-year-old age group [24]. In addition, this study showed that the effect of Asian dust with short-term PM exposure was greater in the BMI < 25 group than in the BMI ≥25 group. Andersen et al. similarly reported that enhanced air pollution effects were not observed with increasing BMI [25]. On the contrary, Miller et al. showed a stronger association between cardiovascular disease and PM_2.5_ concentration with increasing obesity [26]. However, after stratifying the effects of Asian dust by BMI status exclusively, a higher risk was observed in overweight people (data not provided). Although it is well-known that smoking increases the risk of AMI [27], epidemiologic evidence assessing the effects of PM by smoking status is sparse, owing to inadequate data. The results indicate that past smokers have a higher risk of AMI onset than never smokers and current smokers; however, this difference was not significant. Similar results were reported for smokers in the US, where a range of 8–18% increase in cardiovascular mortality per 10 μm/m^3^ increase in ambient PM was estimated relative to the risk in nonsmokers [28]. These findings suggest that the physiological characteristics and underlying factors of specific populations, such as those based on smoking habit, BMI, or age, likely differed regarding their contribution to Asian dust susceptibility. Furthermore, many complex factors contribute to differences in weather conditions and topography; therefore, the current findings require replication.

Some plausible mechanisms have been suggested for the association of Asian dust with adverse health effects. Zhang et al. reported that the particle sizes in Asian dust range from 1 to 8 μm (median, 3 μm), which indicates relatively small particles in the PM_2.5_ size range [29]. Therefore, PM in Asian dust storms could have a similar pathophysiological mechanism regarding the effects on human health as urban PM can lead to systemic oxidative stress or systemic inflammation. These effects could subsequently induce a cascade reaction within the cardiovascular system [30]. Studies on experimental rats in Japan [31] and Taiwan [32] observed inflammatory- and severe toxicity effects of Asian dust storms on blood pressure and heart rates. However, detailed mechanisms underlying AMI onset due to Asian dust exposure remain unclear; this may be because determining the precise composition of Asian dust is complex [33]. Similarly, desert dust moves microorganisms and bio-particulates, such as fungi, viruses, bacteria, and pollen, as well as related lipid components and protein [34]. Further studies are warranted to determine the relationship between the aforementioned components and the increase in AMI incidence.

To the best of our knowledge, this study is the first to thoroughly investigate the dose-response relationship between visibility and STEMI incidence. The results suggest an approximately negative linear exposure-response curve and indicate that visibility degradation varies according to PM_2.5_ concentrations. Similar results were reported by Huang et al. in Shanghai, China [35], who were the first to graphically present the exposure-response relationship between visibility and cardiovascular mortality and show that the exposure-response curve exhibits a negative linear relationship. Some studies assumed an exponential relationship between PM_2.5_ and visibility, with higher PM_2.5_ concentrations corresponding to lower visibility [36]. These studies support a relationship between visibility and PM_2.5_ and PM_10_ concentrations and thus clarify the relationship between visibility and cardiovascular events.

This study exhibits a number of strengths. First, a long study period and a relatively large sample size from the KAMIR database were used; the KAMIR is a prospective, open, observational, multi-center on-line registry on AMI. In contrast, other studies have been limited to a short temporal scope and a small number of patients. Second, arrival time at the hospital due to AMI symptoms was used rather than hospital admission day. Therefore, the data provide novel insights into the mechanisms by which PM exposure may increase risk of AMI associated with Asian dust. Third, estimates from the models were quite robust in relation to inclusion of the weather variables, the use of interaction terms between the daily means of visibility and humidity, and varying lagged structures of PM.

However, this study equally has a number of limitations. First, the ambient air pollution concentration was used as proxy exposure from an air pollution monitoring site rather than personal exposure levels. However, a previous study reported that outdoor air pollution exposure and personal PM_2.5_ exposure seemed particularly problematic, as personal exposure can widely vary depending on lifestyle choices, such as time-activity patterns of individuals, occupational characteristics, and residential environment [37]. Although previous time-series studies used a common approach that relied on exposure data from a single monitoring station to reflect outdoor pollution in the study area, exposure data from 27 monitoring stations spread geographically across Seoul were used, computed by averaging the daily mean concentrations across all stations. In this study, systematic differences between areas in the measurement of covariates. Second, data concerning the chemical and biological composition of Asian dust were not included. Accordingly, a further study including the composition and toxicity of Asian dust is necessary.

## Conclusions

This study provides new insights regarding the effects of PM on AMI during Asian dust days. These results suggest that it is crucial to reduce PM_2.5_ and PM_10_ emissions, particularly during Asian dust days, and this would reduce the incidence of STEMI and AMI among young patients. Identifying modifier effects of PM could provide knowledge for future risk assessment studies.

## Supplementary Information

**Additional file 1.** Correlations between exposure variables during 2003–2013 in Seoul.

**Additional file 2.** Relative risks of AMI per 10 μg/m^3^ increase in current-day PM_2.5_ and PM_10_ concentrations by subgroup with single lags.

**Additional file 3.** Relative risk (RR) of Asian dust associated with acute myocardial infarction, stratified by age and body mass index (BMI) after adjusting for PM_10._

**Additional file 4.** Association between Asian dust and acute myocardial infarction by smoking status, after adjustment for PM_2.5_ by lag days.

**Additional file 5.** Relative risk (RR) of Asian dust associated with ST elevation myocardial infarction after adjustment for PM_2.5_ with single lag days (from current day to the previous 6 days): results of the main analyses models and sensitivity analyses models.

## Data Availability

The data that support the findings are available from [Korea Centers for Disease Control and Prevention] but restrictions apply to the availability of these data, which were used under license for the current study, and so are not publicly available. Data are however available from the authors upon reasonable request and with permission of [Korea Centers for Disease Control and Prevention].

## Abbreviations

AMI: Acute myocardial infarction
PM: Particulate matter
MI: Myocardial infarction
STEMI: ST-elevation MI
NSTEMI: Non ST-elevation MI
RR: Relative risk
CI: Confidence interval
KAMIR: Korea Acute Myocardial Infarction Registry
BMI: Body mass index
