# Supplementary material for: Effects of Asian dust-derived particulate matter on ST-elevation myocardial infarction: retrospective, time series study
Source: BMC Public Health. 2021 Jan 7;21:68. doi: 10.1186/s12889-020-10067-y (PMC7791846; doi:10.1186/s12889-020-10067-y)
Supplement: Supplementary file 1 — Additional file 1. Correlations between exposure variables during 2003–2013 in Seoul. [file 12889_2020_10067_MOESM1_ESM.pdf]

Correlations between exposure variables in Seoul, during 2003–2013.

	$PM_{2.5}$	PM_{10}	NO_2	SO_2	O_3	CO	Temperature	Humidity	Visibility	Visibility[b]
$PM_{2.5}$	1.00	0.83**	0.63**	0.64**	0.04*	0.66**	-0.11**	0.04*	-0.66**	-0.70**
PM_{10}		1.00	0.52**	0.57**	0.04*	0.56**	-0.18**	-0.1**	-0.51**	-0.53**
NO_2			1.00	0.66**	-0.23**	0.80**	-0.25**	-0.12**	-0.37**	-0.43**
SO_2				1.00	-0.17**	0.75**	-0.42**	-0.21**	-0.29**	-0.34**
O_3					1.00	-0.38**	0.56**	-0.11**	0.03	0.02
CO						1.00**	-0.42**	-0.04*	-0.42**	-0.43**
Temperature							1.00	0.40**	-0.08**	0.02
Humidity								1.00	-0.58**	-0.33**
Visibility									1.00	

Significant at *$p < 0.05$.

Significant at **$p < 0.001$.

[a]When visibility was less than 10 km

[b]The visibility value was excluded when relative humidity was higher than 80%.
