# Supplementary material for: Effects of Asian dust-derived particulate matter on ST-elevation myocardial infarction: retrospective, time series study
Source: BMC Public Health. 2021 Jan 7;21:68. doi: 10.1186/s12889-020-10067-y (PMC7791846; doi:10.1186/s12889-020-10067-y)
Supplement: Supplementary file 2 — Additional file 2. Relative risks of AMI per 10 μg/m3 increase in current-day PM2.5 and PM10 concentrations by subgroup with single lags. [file 12889_2020_10067_MOESM2_ESM.pdf]

Relative risks of AMI per 10 μg/m^3 increase in current-day $PM_{2.5}$ and PM_{10} concentrations by subgroup with single lags.

	$PM_{2.5}$	PM_{10}
Total	1.001 (1.000-1.001)	1.0003 (1.000-1.001)
STEMI	1.001 (0.999, 1.003)[a]	1.000 (0.999, 1.001)
NSTEMI	1.002 (0.999, 1.004)	1.001 (1.001, 1.002)*
Under 65[a]	1.0002 (0.997, 1.0031)	1.0003 (0.999, 1.0016)
Over 65	1.001 (0.999, 1.003)	1.001 (1.000, 1.002)

[a] Two-day single lags were used for PM2.5 concentration

PM_{10}, particulate matter with an aerodynamic diameter smaller than 10 µm; $PM_{2.5}$, particulate matter with an aerodynamic diameter smaller than 2.5 µm; STEMI, ST-elevation myocardial infarction; NSTEMI, non-ST-elevation myocardial infarction; AMI, acute myocardial infarction.
