# Supplementary material for: Effects of Asian dust-derived particulate matter on ST-elevation myocardial infarction: retrospective, time series study
Source: BMC Public Health. 2021 Jan 7;21:68. doi: 10.1186/s12889-020-10067-y (PMC7791846; doi:10.1186/s12889-020-10067-y)
Supplement: Supplementary file 3 — Additional file 3. Relative risk (RR) of Asian dust associated with acute myocardial infarction, stratified by age and body mass index (BMI) after adjusting for PM10. [file 12889_2020_10067_MOESM3_ESM.pdf]

Figure S1. Relative risk (RR) of Asian dust associated with acute myocardial infarction, stratified by age and body mass index (BMI) after adjusting for PM_{10}.

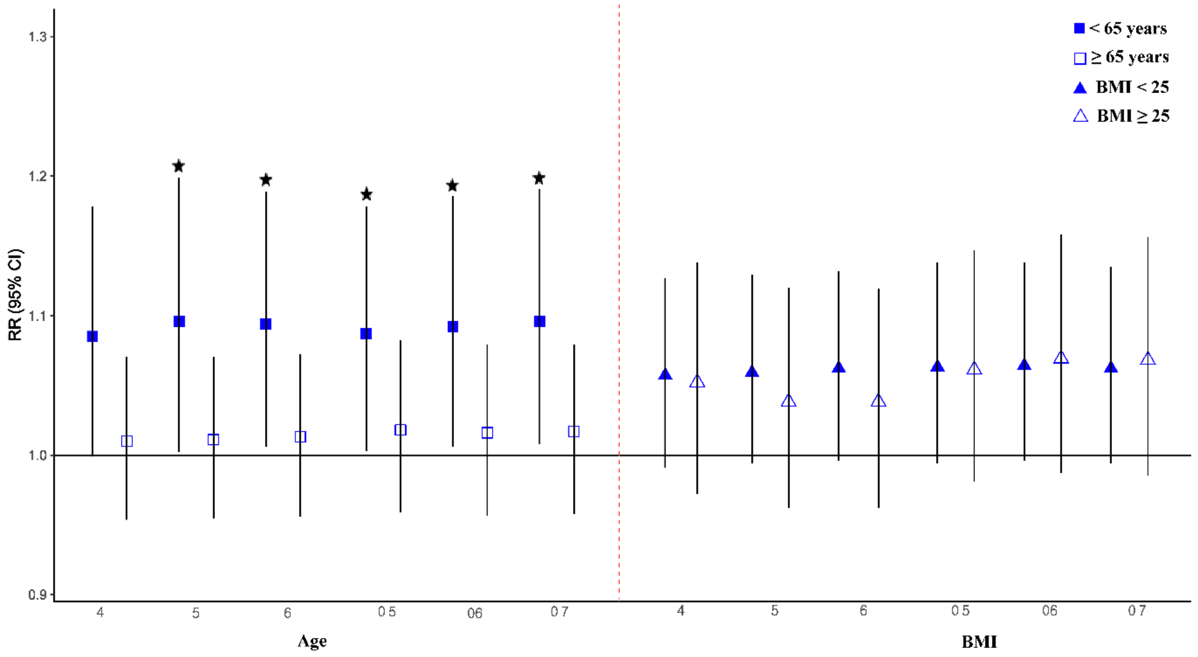

RRs were adjusted for long-term trends, seasonality, two-day moving average of temperature, sea level pressure, and relative humidity. Six-day (lag 6) lags and two-day (lag 2) lags of Asian dust were used for those aged <65 and ≥65 years, respectively. Two-day (lag 2) lags and five-day (lag 5) lags of Asian dust were used for those with BMI<25 and ≥25 kg/m^2, respectively.

* Significant at $p < 0.05$.
