# Supplementary material for: Effects of Asian dust-derived particulate matter on ST-elevation myocardial infarction: retrospective, time series study
Source: BMC Public Health. 2021 Jan 7;21:68. doi: 10.1186/s12889-020-10067-y (PMC7791846; doi:10.1186/s12889-020-10067-y)
Supplement: Supplementary file 4 — Additional file 4. Association between Asian dust and acute myocardial infarction by smoking status, after adjustment for PM2.5 by lag days. [file 12889_2020_10067_MOESM4_ESM.pdf]

Association between Asian dust and acute myocardial infarction by smoking status, after adjustment for $PM_{2.5}$ by lag days.

Lag days	Never smoking RR (95% CI)	Past smoking RR (95% CI)	Current smoking RR (95% CI)
4	1.041 (0.956–1.133)	1.096 (0.979–1.227)	1.052 (0.988–1.122)
5	1.043 (0.959–1.136)	1.138 (0.997–1.298)	1.069 (0.993–1.151)
6	1.042 (0.958–1.135)	1.113 (0.989–1.253)	1.058 (0.990–1.130)

RR, relative risk; CI, confidence interval; $PM_{2.5}$, particulate matter with an aerodynamic diameter smaller than 2.5 μm
