# Supplementary material for: Effects of Asian dust-derived particulate matter on ST-elevation myocardial infarction: retrospective, time series study
Source: BMC Public Health. 2021 Jan 7;21:68. doi: 10.1186/s12889-020-10067-y (PMC7791846; doi:10.1186/s12889-020-10067-y)
Supplement: Supplementary file 5 — Additional file 5. Relative risk (RR) of Asian dust associated with ST elevation myocardial infarction after adjustment for PM2.5 with single lag days (from current day to the previous 6 days): results of the main analyses models and sensitivity analyses models. [file 12889_2020_10067_MOESM5_ESM.pdf]

Relative risk (RR) of Asian dust associated with ST-elevation myocardial infarction after adjustment for $PM_{2.5}$ with single lag days (from current day to the previous six days): results of the main analyses models and sensitivity analyses models.

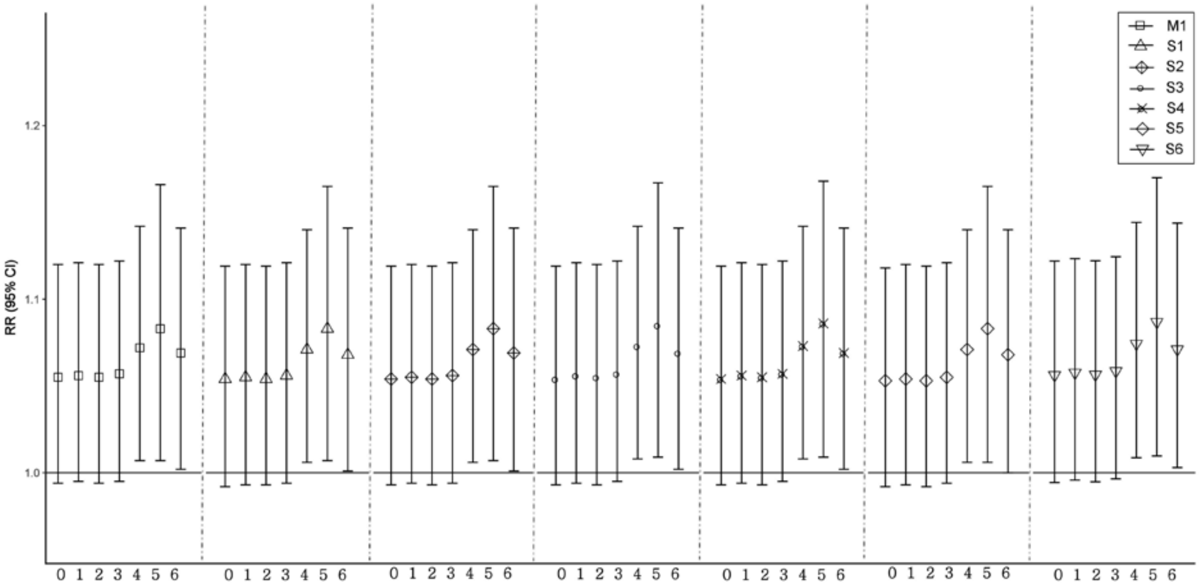

RRs for the main model were adjusted for six days (lag 6) lags of Asian dust, two-day moving average (lag 0–1) of temperature, sea level pressure, relative humidity, long-term trends, and seasonality. M1, main results; S1, use of interaction terms between daily mean of visibility and humidity (higher than 70%); S2, use of interaction terms between daily mean of visibility and humidity (higher than 80%); S4, change of three-day (lag 0–2) moving average of temperature; S5, change of four-day (lag 0–3) moving average of temperature; S5, df = 8 was used for temporal trends; S6, df = 9 was used for temporal trends. $PM_{2.5}$, particulate matter with an aerodynamic diameter smaller than 2.5 µm
